# Exploring miRNA Signature and Other Potential Biomarkers for Oligometastatic Prostate Cancer Characterization: The Biological Challenge behind Clinical Practice. A Narrative Review

**DOI:** 10.3390/cancers13133278

**Published:** 2021-06-30

**Authors:** Giulia Corrao, Mattia Zaffaroni, Luca Bergamaschi, Matteo Augugliaro, Stefania Volpe, Matteo Pepa, Giuseppina Bonizzi, Salvatore Pece, Nicola Amodio, Francesco Alessandro Mistretta, Stefano Luzzago, Gennaro Musi, Sarah Alessi, Francesco Maria La Fauci, Chiara Tordonato, Daniela Tosoni, Federica Cattani, Sara Gandini, Giuseppe Petralia, Gabriella Pravettoni, Ottavio De Cobelli, Giuseppe Viale, Roberto Orecchia, Giulia Marvaso, Barbara Alicja Jereczek-Fossa

**Affiliations:** 1 Division of Radiation Oncology, IEO, European Institute of Oncology IRCCS, Via Ripamonti 435, 20141 Milan, Italy; giulia.corrao@ieo.it (G.C.); mattia.zaffaroni@ieo.it (M.Z.); luca.bergamaschi@ieo.it (L.B.); stefania.volpe@ieo.it (S.V.); matteo.pepa@ieo.it (M.P.); giulia.marvaso@ieo.it (G.M.); barbara.jereczek@ieo.it (B.A.J.-F.); 2 Department of Oncology and Hemato-Oncology, University of Milan, 20141 Milan, Italy; salvatore.pece@ieo.it (S.P.); Gennaro.Musi@ieo.it (G.M.); chiara.tordonato@ieo.it (C.T.); giuseppe.petralia@ieo.it (G.P.); gabriella.pravettoni@ieo.it (G.P.); ottavio.decobelli@ieo.it (O.D.C.); giuseppe.viale@ieo.it (G.V.); 3 Department of Pathology, IEO, European Institute of Oncology IRCCS, 20141 Milan, Italy; giuseppina.bonizzi@ieo.it; 4 Novel Diagnostics Program, IEO, European Institute of Oncology IRCCS, 20141 Milan, Italy; daniela.tosoni@ieo.it; 5 Department of Experimental and Clinical Medicine, Magna Graecia University of Catanzaro, 88100 Catanzaro, Italy; amodio@unicz.it; 6 Department of Urology, IEO, European Institute of Oncology IRCCS, 20141 Milan, Italy; FrancescoAlessandro.Mistretta@ieo.it (F.A.M.); stefano.luzzago@ieo.it (S.L.); 7 Division of Radiology, IEO, European Institute of Oncology IRCCS, 20141 Milan, Italy; sarah.alessi@ieo.it; 8 Unit of Medical Physics IEO, European Institute of Oncology IRCCS, 20141 Milan, Italy; francesco.lafauci@ieo.it (F.M.L.F.); federica.cattani@ieo.it (F.C.); 9 Department of Experimental Oncology, IEO, European Institute of Oncology IRCCS, 20141 Milan, Italy; sara.gandini@ieo.it; 10 Applied Research Division for Cognitive and Psychological Science, IEO, European Institute of Oncology IRCCS, 20141 Milan, Italy; 11 Scientific Direction, IEO, European Institute of Oncology IRCCS, 20141 Milan, Italy; roberto.orecchia@ieo.it

**Keywords:** oligometastatic prostate cancer, biomarker, miRNA, CTC, epi-miRNA

## Abstract

**Simple Summary:**

The oligometastatic prostate cancer state is defined as the presence of a number of lesions ≤ 5 and has been significantly correlated with better survival if compared to a number of metastases > 5. In particular, patients in an oligometastatic setting could benefit from a metastates directed therapy, which could control the disease delaying the start of systemic therapies. For this reason, the selection of true-oligometastatic patients who could benefit from such approach is particularly important in this setting. The aim of the present narrative review is to report the current state of the art on the liquid biopsy-derived analytes and their reliability as biomarkers in the clinics for the identification of true-oligometastatic patients. This kind of molecular profiling could refine current developments in the era of precision oncology allowing patients’ stratification and leading to more refined therapeutic strategies.

**Abstract:**

In recent years, a growing interest has been directed towards oligometastatic prostate cancer (OMPC), as patients with three to five metastatic lesions have shown a significantly better survival as compared with those harboring a higher number of lesions. The efficacy of local ablative treatments directed on metastatic lesions (metastases-directed treatments) was extensively investigated, with the aim of preventing further disease progression and delaying the start of systemic androgen deprivation therapies. Definitive diagnosis of prostate cancer is traditionally based on histopathological analysis. Nevertheless, a bioptic sample—static in nature—inevitably fails to reflect the dynamics of the tumor and its biological response due to the dynamic selective pressure of cancer therapies, which can profoundly influence spatio-temporal heterogeneity. Furthermore, even with new imaging technologies allowing an increasingly early detection, the diagnosis of oligometastasis is currently based exclusively on radiological investigations. Given these premises, the development of minimally-invasive liquid biopsies was recently promoted and implemented as predictive biomarkers both for clinical decision-making at pre-treatment (baseline assessment) and for monitoring treatment response during the clinical course of the disease. Through liquid biopsy, different biomarkers, commonly extracted from blood, urine or saliva, can be characterized and implemented in clinical routine to select targeted therapies and assess treatment response. Moreover, this approach has the potential to act as a tissue substitute and to accelerate the identification of novel and consistent predictive analytes cost-efficiently. However, the utility of tumor profiling is currently limited in OMPC due to the lack of clinically validated predictive biomarkers. In this scenario, different ongoing trials, such as the RADIOSA trial, might provide additional insights into the biology of the oligometastatic state and on the identification of novel biomarkers for the outlining of true oligometastatic patients, paving the way towards a wider ideal approach of personalized medicine. The aim of the present narrative review is to report the current state of the art on the solidity of liquid biopsy-related analytes such as CTCs, cfDNA, miRNA and epi-miRNA, and to provide a benchmark for their further clinical implementation. Arguably, this kind of molecular profiling could refine current developments in the era of precision oncology and lead to more refined therapeutic strategies in this subset of oligometastatic patients.

## 1. Introduction

Prostate cancer (PCa) is the most commonly diagnosed solid-organ malignancy and the second leading cause of cancer death in men worldwide [1]. Despite the high long-term survival in localized PCa, metastatic PCa remains largely associated with an overall low survival rate [2].

Recently, a growing interest is directed towards oligometastatic prostate cancer (OMPC), as the presence of a number of lesions ≤ 5 has been significantly correlated with better survival if compared to a number of metastases > 5 [3]. The *oligometastatic* concept was introduced for the first time by Hellman and Weichselbaum in 1995 to describe an intermediate phase between localized disease and extensive metastatic state [4]. To date, there is still no univocal definition of the oligometastatic state, even if in the scientific community, it is commonly considered as the presence of up to 3–5 lesions [5,6]. It is necessary to distinguish between two conditions of OMPC: synchronous OMPC (metastases that are already detectable at the initial diagnosis of primary tumor) and metachronous OMPC (metastases that are detected or become clinically evident at later stages of the disease course after initiation of treatment of the primary tumor treatment). Our review examines the latter scenario.

The efficacy of local ablative treatments directed towards metastatic lesions (metastases directed treatments—MDTs) in patients with OMPC was extensively investigated, with the aim of preventing the systemic spread of the disease and delaying the start of systemic androgen deprivation therapies (ADT) [1,7,8,9,10,11,12,13,14]. Nevertheless, the use of MDTs is still controversial due to the paucity of prospective randomized efforts. Notably, the publication of results from the SABR-COMET, STOMP and ORIOLE trials has fostered further research in the field, and upcoming evidence is likely to further modify the treatment scenario for this subset of patients [15,16,17]. In this perspective, ongoing studies such as the RADIOSA trial, a randomized phase II clinical trial [18] aiming to compare stereotactic body radiotherapy (SBRT) alone and in combination with 6 months ADT for the treatment of oligorecurrent-castration-sensitive-PCa (OCS-PCa), could foster the use of MDT in a selected subset of PCa patients.

With the emerging use of whole-body magnetic resonance imaging (WB-MRI) and Ga-prostate-specific membrane antigen (PSMA) positron emission tomography/computed tomography (PET/CT), it has become easier to detect the presence of metastases in patients with early biochemical recurrence [19,20,21,22,23,24]. Despite the accuracy of these investigations, it is likely that a number of patients who are already polimetastatic will escape detection, especially at low prostate-specific antigen (PSA) levels, with negative consequences on correct clinical choices [25,26].

Since the oligometastatic state has a peculiar behavior as compared with heavy burden disease, the existence of distinct underlying biological and molecular mechanisms was hypothesized [27]. The use of PSA alone, due to its poor specificity, seems to be increasingly limited in discerning the different categories of PCa patients; therefore, the identification of other markers could provide additional information to individuate the correct prognosis and consequently propose the best treatment course, especially in OMPC [28]. Ideally, these analytes should be easily obtainable in a non-invasive manner, easy to implement across facilities, reproducible and as inexpensive as those that can be collected from serum.

For these reasons, the identification of novel biomarkers could potentially improve the treatment of advanced PCa by identifying selected oligometastatic patients who could benefit from MDT. The aim of the present review, which represents an ancillary study of the RADIOSA trial [18], is to investigate the state of the art biology behind the OMPC and possible candidate molecular markers for the identification of true oligometastatic patients eligible for MDT. Special focus is paid to oligorecurrent patients diagnosed with metachronous oligometastases after definitive treatment on the primary tumor.

## 2. Polimetastatic versus Oligometastatic PCA 

### 2.1. Burden of Disease

The burden of disease in metastatic PCa represents a fundamental prognostic factor [29], so the possibility to discern between different burdens of systemic disease represents an area of growing interest, with the final aim of personalizing therapeutic approaches

The distinction between high and low burden of disease in PCa comes from the CHAARTED trial [30], a prospective randomized trial for patients affected by hormone-sensitive metastatic PCa. In this trial, the high-volume disease was defined as the presence of ≥4 visceral and/or bone metastases with at least one outside of the vertebral column and pelvis.

Based on CHAARTED trial results, subsequent scientific research supported the initiation of systemic therapies in the case of high-burden disease and of local therapies in low-burden disease [15,16]. In fact, in patients with a low metastatic burden, ablative radiotherapy of metastatic lesions is believed to result in an advantage in terms of disease-free survival and overall survival and in an improvement in quality of life by reducing as much as possible the side effects of systemic therapeutic intervention.

However, probably due to the underestimation of the real metastatic burden with the standard imaging methods, almost 30% of patients treated with MDT experiment a rapid progression to polymetastatic prostate cancer (PMPC) within 1 year [31].

### 2.2. Biological Differences

A first hypothesis argues for a molecular similarity between OMPC and PMPC, considering OMPC merely as an earlier detected metastatic disease state; an alternative hypothesis supports the dual clonal origin of OMPC and PMPC due to the existence of clones of tumor cells with heterogeneous genetic make-up. Recent available data seem to favor the latter, supporting intense efforts to distinguish true OMPC from PMPC based on the use of clinicopathological parameters and other biomarkers in combination with imaging methods already used in clinical practice [32,33].

The oligometastatic state is recently believed to be a phenotype with an underdeveloped metastatic potential and an inherently slow natural history. In particular, OMPC is considered as an intermediate state between localized PCa and PMPC, with probable differences in genetic and molecular background [34].

The conversion to PMPC is a multistep process promoted by both genetic and epigenetic molecular alterations, involving a consequent series of well-known cellular events: (i) loss of cellular adhesion; (ii) increased motility and invasiveness; (iii) entering the circulation; (iv) invasion and proliferation in organs, a number of cellular adaptations that are part of the broader repertoire of epithelial-to-mesenchymal transition phenotypes [35,36,37].

Besides other factors such as cytokines and exosomes, metastatic progression seems to also rely on the communication between cancer cells and their local and distant environment through micro Ribo-Nucleic Acids (miRNAs), which post-transcriptionally control gene expression and regulate several steps in the metastatic cascade [38]. Furthermore, there are several ways in which metastatic cells originate: (i) synchronous seeding (from the primary tumor), (ii) metachronous seeding (from other metastases) and (iii) self-seeding (return to the site of origin) [39].

Although OMPC represents a spread of tumor cells outside the primary localization, the clone of malignant cells probably does not have the biological requirements to disseminate in multiple sites throughout the body [40,41]. While these cells have the capability to evade the immune system and cause isolated metastases, they lack some classic features of malignancy, with a consequent lower metastatic potential, including (i) resistance to cell death; (ii) avoidance of immune destruction; (iii) replication immortality [42,43].

In consideration of these assumptions, it is reasonable to approach OMPC with local ablative treatments, avoiding aggressive systemic therapies. The stratification of metastatic PCa patients and the subsequent identification of the subset of patients who can really benefit from MDTs, using both new specific biomarkers and current imaging methods, could represent an intriguing future strategy.

## 3. Benefit from Metastases-Directed Therapy (MDT)

For newly diagnosed oligorecurrent PCa, recently available data suggest the administration of local ablative treatments to all the visible metastases. This therapeutic strategy aims to postpone the start of systemic therapy.

As mentioned above, it was proven that the treatment of macrometastases reduces the risk of progressive metastasis-to-metastasis seeding, even in men with a controlled primary tumor [44,45]. However, MDT is still not the standard of care in OMPC, and more prospective trials are needed to allow its full implementation into clinical practice [46].

To the best of our knowledge, data from three prospective studies on MDT for oligorecurrent PCa were available at the time of writing [16,17,47].

In addition, a recent metanalysis explored the role of SBRT in the setting of oligorecurrent PCa. The study comprehends six studies (two randomized and four observational works) and a total of 396 patients treated with SBRT. The results of the study showed that patients in an oligometastatic setting might benefit from SBRT both in terms of local and biochemical control.

The rationale behind this benefit originates from the assumption that specifically hypofractionated treatments have some novel systemic cell mechanisms, including tumor vascular disruption and increased immune reactivity [48]. 

Surgical resection represents an alternative approach to MDT. Between studies that explored the efficacy and the safeness of MDT in oligorecurrent PCa, some included surgery as a possible strategy. In particular, in the STOMP [16] trial, clinicians could administer SBRT as well as surgery with ablative intent, and six patients underwent surgery.

Pelvic lymph nodes are a very common site of recurrence, and lymph node dissection is an elective surgical procedure. In order to limit side effects correlated to this approach and thanks to the advent of next-generation imaging techniques, new surgical strategies are emerging, such as the use of pre-operative hook-wire localization under CT guidance of lymph node detected by PSMA PET [49].

Despite progress, SBRT maintains many advantages over surgery, such as the less invasive procedure, lower side effects, and potentially more cost-effective. However, surgery may be preferred in relation to tumor location (central lung lesions, large brain metastases).

In this scenario, the RADIOSA trial, a randomized phase II clinical trial, is currently ongoing in the recruiting phase [18]. The primary aims are to compare SBRT +/− ADT for OCS-PCa in terms of efficacy, toxicity and quality of life (QoL) and to develop biology and imaging-based prognostic tools in order to discriminate OCS-PCa subclasses better.

With an estimated recruiting of 160 OCS-PCa patients in 3 years, a sufficient number of blood samples will be collected to perform comprehensive biological patient profiling and possibly provide further insights into the biology underlying oligometastatic state.

## 4. Emerging Biomarkers for the Identification of True Oligometastatic Patients Eligible for MDT

### 4.1. Liquid Biopsy and Next Generations Sequencing (NGS)

Definitive diagnosis of PCa is traditionally based on histopathological analysis. Nevertheless, due to the multifocal nature of most PCas [50], as the tumor progresses, the static result from a biopsy sample will become even more inadequate to reflect dynamics of tumor evolution and its underlying biological modifications under the selective pressure of cancer therapies. These difficulties can be ascribed to the intrinsic difficulties in obtaining biopsy samples from metastases and to the inability to perform selective genetic tests on biopsy-derived tissue. Furthermore, complications may also arise from the invasive nature of biopsy procedures, which make not all cancer patients eligible for surgery due to their intrinsic fragility.

Nowadays, even with new imaging technologies allowing an increasingly early detection, the diagnosis of oligometastasis is currently based exclusively on traditional radiological investigations. Nevertheless, objective categorization of true oligometastatic patients from the ones with a trend to progress to poly-metastatic patients relies on the profile of the biological behavior of the tumor; for this purpose, a minimally invasive real-time monitoring method could be beneficial for both patients and clinicians. This could avoid expensive treatments with limited clinical benefit and potential associated toxicity or, alternatively, provide a group of oligometastatic patients with curative treatment.

Liquid biopsy has recently emerged as a promising minimally invasive approach allowing to overcome the static bioptic approach and to reflect the dynamic tumor modifications over time, specifically those involving its genomic evolution [50,51]. Through liquid biopsy, different biomarkers, commonly extracted from blood, urine or saliva, can be characterized, including circulating tumor cells (CTCs), circulating cell-free DNA (cfDNA) such as circulating tumor DNA (ctDNA), miRNA and exosomes [52,53].

However, the utility of tumor profiling is currently limited in MPC due to the lack of validated predictive biomarkers [54]. In this scenario, liquid biopsies have the potential to act as a tissue substitute and cost-efficiently accelerate trials designed to identify predictive biomarkers.

A significant advancement in the development of personalized treatments according to the tumor’s molecular profile was facilitated by the rapid development of next-generation sequencing (NGS) technologies.

While Sanger sequencing in the past could satisfy the clinical request of single-gene testing, the advent of more innovative and sensitive approaches fostered the discovery of multiple genetic alterations to be implemented as predictive biomarkers for clinical use [55].

NGS is a high-throughput technology based on a “sequence biosynthesis” principle [56] and, unlike Sanger sequencing, allows for the analysis of a huge amount of sequences in parallel, generating high-throughput data from both different sequencing positions and from different patients simultaneously.

A typical NGS workflow requires the following steps: library preparation, amplification, sequencing, and data analysis. Of note, an in-house validation in any phase of sequencing workflow is required before implementation in a clinical diagnostic routine. This validation process is required not only for the development of different assays and gene panels but also for the bioinformatics pipeline. A more specialized review on NGS is recommended to the readers [57].

Compared to other common transcript quantification methods, such as real-time polymerase chain reaction (PCR), the implementation of NGS in clinical practice brings an increased analytical and clinical sensitivity in the identification of single nucleotide polymorphisms (SNPs) and other genetic alteration in a wild type background.

In addition, although PCR-based methods are sensitive and inexpensive, they can only screen for known genetic variants, while NGS has the possibility to screen for unknown variants.

Another important consideration is related to the choice of different commercially available gene panels differentiated for clinical purposes, from clinical trials recruiting (10 to 50 genes panels) to the analysis of the whole spectrum of cancer-related genes (>400 genes panels) [58,59].

Among different NGS approaches, chromatin immunoprecipitation analysis (ChIP-seq) represents an area of emerging interest. A ChIP-seq analysis aims to locate the DNA binding site of any protein and, therefore, investigates epigenetic factors that affect gene expression. This can help uncover the interaction pattern of elements such as transcription factors, structural proteins, or any chromatin-associated protein and gives insights into the epigenetic mechanisms that guide cell differentiation and tumor or metastasis development.

### 4.2. Circulating Tumor Cells (CTCs)

CTCs are cancer cells originating from macroscopic tumor sites (either primary or metastases) and released into the bloodstream. Here, CTCs could be found as single CTCs or CTC clusters, with the latter being more often associated with a higher metastatic potential [60,61].

Since CTCs may reflect the current tumor status, there is a growing interest in identifying genomic alterations in CTCs that could aid the decision workflow in targeted therapies.

A recent study by Gkontela et al. [61] provided insights about how CTC clusters intrinsic differences have a direct impact on the DNA methylation status and thus influence important regulatory regions related to cancer proliferation, suggesting that agents disrupting these clusters could suppress spontaneous metastatic formations.

A 2020 study by Faugeroux et al. [62] emphasized the potential of CTCs in representing metastases mutational content and tumor diversity that would be otherwise inaccessible. Therefore, by offering real-time monitoring of a constantly evolving disease and detecting potentially critical SNPs via liquid biopsy, CTC sequencing can serve an unmet need for optimal therapy selection and precision medicine.

A study by Mandel et al. [63] reported pre- and post-operative CTC enumeration in OMPC patients treated with cytoreductive prostatectomy. The results showed that CTC enumeration both at diagnosis and at 6 months was superior to common biomarkers, such PSA, as a prognostic factor for oncological outcomes. Notwithstanding the low statistical sample (33 patients), the study points to the potential of CTC as a valuable biomarker in OMPC.

A significant barrier in the study of CTCs is their relative rarity in the bloodstream with respect to other cells. Therefore, CTCs isolation results as a critical step for their validation as a candidate biomarker. One possible approach is based on immunomagnetic isolation: being CTCs mostly of epithelial origin, they express epithelial cellular adhesion molecule (EpCAM) antigens on the cell membrane, while the background of blood cells does not. A disadvantage of this method is that not all CTCs express EpCAM and that its expression is not always easily detectable and, therefore, part of the CTC population may be lost [64,65]. Another important limitation in this setting is represented by the fact that the levels of EpCAM in cancer stem cells could be lower than in the bulk tumor mass, and therefore CTCs would not display cancer stem cells.

Another isolation method is based on background cell depletion by bead-antibodies against CD45 and CD15, which are not expressed by CTCs [66].

An alternative method for CTCs detection is based on the fact that telomerase expression, characteristic of cancer, and, specifically, the catalytic subunit of human telomerase, namely telomerase reverse transcriptase (hTERT), is mutated in normal human somatic cells but is active in most cancers. This method uses replication-competent adenovirus regulated by hTERT promoter to detect CTCs [67].

However, none of these approaches for CTCs enrichment guarantees a pure and complete population of tumor cells; therefore, a detection method to distinguish CTCs from the background is essential to consistently include CTCs as valuable predictive biomarkers in clinical routine [65].

In this scenario, it was speculated that a combination of methods based on different properties could solve most of the aforementioned issues.

Since each of the above-mentioned techniques has intrinsic limitations, and none is robust enough to be considered as the best one, it was proposed that the combination of different approaches can be helpful to solve most of the issues mentioned above [68]. As of today, great efforts are still necessary to overcome biological problems related to the study of the circulating tumor population, as well as to increase the biological knowledge and clarify the clinical role of CTCs in solid cancer patients.

### 4.3. Circulating Cell-Free DNA (cfDNA)

cfDNA, or ctDNA, shed from apoptotic and necrotic cells, comprises both genomic and mitochondrial DNA and can be used as a biomarker to characterize the mutational and epigenomic status in advanced solid tumors [69]. The ctDNA concentration in plasma was correlated with both tumor size and clinical stage of the malignancy [70,71]. Additionally, the half-life of these molecules is relatively short (1–2 h), which provides real-time insight into the tumor status. Clinical studies showed that healthy individuals present lower cfDNA levels, indicating a relatively simple analysis involving the mere cfDNA quantification as a valuable biomarker [72,73]. The exact measure of cfDNA can be challenging due to the high fragmentation degree and the overall low concentration. The main source of cfDNA is also controversial. In fact, while the serum presents a higher concentration of cfDNA molecules, serum-derived samples are often contaminated by a clotting process, and therefore plasma is actually considered a more valuable cfDNA source despite the lower overall concentration [74,75].

As the total cfDNA increases with the tumor growth, it was hypothesized that cfDNA derives directly from living tumor cells and that CTCs could be an alternative cfDNA source [76].

In the ongoing ORIOLE trial [17], ctDNA collected at baseline from 54 participants was profiled by deep sequencing, and no significant differences in ctDNA concentration were detected between participants according to their progression status. Additionally, even though PFS resulted significantly longer among participants receiving SBRT with respect to those in the observation arm, such advantage was not confirmed in a subgroup analysis considering the high-risk mutation status.

Although it appears a promising biomarker, recent reports have shed light on some inaccuracies of commercial laboratory cfDNA testing in patients with PCa [77]. Therefore, further efforts to enhance the accuracy and reproducibility of cfDNA detection methods are needed to really exploit the clinical benefit of cfDNA and ctDNA in a personalized medicine scenario.

### 4.4. Exosomes

It was speculated that a better understanding of the determinants of oligometastases could come from molecular studies on the signaling between the primary tumor and its metastatic sites. Exosomes are nanoscale extracellular vesicles that have a role in the exchange of genetic material, implicated in tumor cell growth and invasion, favoring disease dissemination by creating a pro-tumor micro-environment and the creation of premetastatic niches [78,79,80,81].

By analyzing the exosome proteins derived from PCa cells, the researchers found a high level of molecules stimulating tumor cell migration and metastases, such as the b4 and avb6 integrins, vinculin and the Trop-2 transmembrane glycoprotein [82,83]. In addition, cancer-derived exosomes can promote EMT through miRNAs, which play an important role in the conversion from benign to malignant cancers and in the regulation of the response to docetaxel, such as miR-34 in prostate cancer cells and cell-derived exosomes targeting Bcl-2 [84].

On the basis of these findings, the role of exosomes in the early phases of tumor metastatization seems to make them interesting and worth to be explored biomarkers for future diagnostic approaches in the oligometastatic setting.

## 5. Micro Ribo-Nucleic Acid (miRNA)

Among emerging analytes to define disease biology, RNA-based biomarkers display several advantages over those relying on DNA. Firstly, the expression of RNA molecules is highly tissue- and disease-specific. Therefore, modifications in RNA expression directly reflect the changes within the cancer cells. Secondly, an RNA-based approach allows for an investigation of the families of the non-coding RNAs [53].

miRNAs are short non-coding transcripts of 17–25 nucleotides, which participate in gene regulation at a post-transcriptional level. Since a single miRNA can target hundreds to thousands of mRNAs [85], it is obvious that miRNAs can regulate several complex signaling pathways. Currently, more than 4800 mature human miRNAs are recorded in the miRBase v22 [86]. Since the tumor tissue usually releases those transcripts inside exosomes, liquid biopsy results particularly suitable for miRNA analysis.

**Figure 1:**
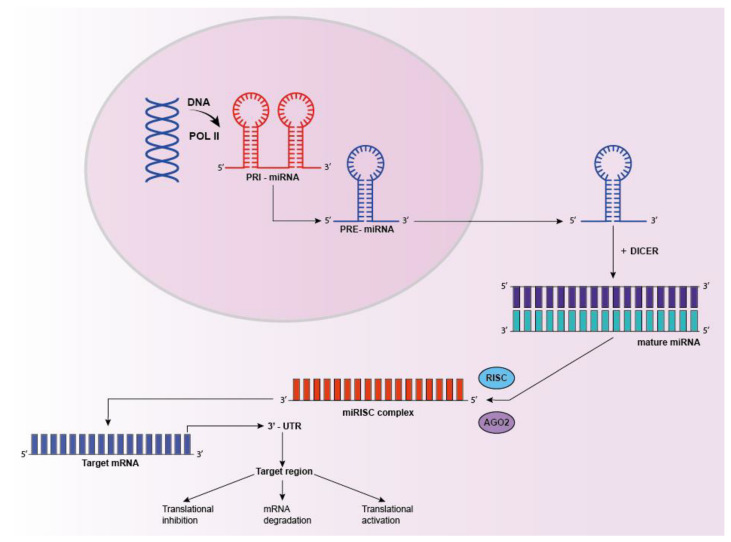
miRNA processing. miRNAs are transcribed in the nucleus as primary transcripts (pri-miRNAs), processed in pre-miRs, exported into the cytoplasm and subsequently processed by multi-protein complex DICER to generate mature duplex miRNAs. One strand of the mature miRNA (guide strand) is loaded into the miRISC complex to target mRNA by sequence complementarity. This interaction results in gene suppression by targeted mRNA degradation, translational repression or translational activation in processing bodies.

miRNA genes are transcribed by RNA polymerase II-III into long primary transcripts (pri-miRNAs), which are further cleaved into shorter pre-miRNAs. Pre-miRNA is then exported out of the nucleus, processed by enzyme DICER (RNase III endonuclease) into a mature miRNA duplex. To guarantee the interaction with 3′ UTR of their targets mRNAs, mature miRNAs must be loaded onto the Argonaute proteins (AGO), forming the central core of the RNA-induced silencing complex (RISC). Within the RISC complex, miRNAs induce silencing by both target destabilization and/or translational repression. Imperfect binding to the 3′ untranslated region of mRNAs leads to repression of protein translation, while in case of perfect or near-perfect complementarity, miRNAs induce the endonuclease cleavage and mRNAs degradation [87] (Figure 1).

Among non-coding RNAs, miRNA have shown great potential as cancer biomarkers since their first report in the context of malignant diseases [88]. miRNA deregulation is often associated with tumorigenesis (alteration of cell growth, differentiation or apoptotic process) as well as the epithelial–mesenchymal transition (EMT) and metastasis formation [89].

Due to their high stability in biological fluids and the possibility to be detected from a small sample volume, miRNAs are emerging as valuable biomarker candidates for tumor detection. Cheng et al. [90], in a 2018 study, reported evidence that circulating miRNA could act as prognostic biomarkers in patients with metastatic hormone-sensitive PCa. In their trial, they demonstrated that several baseline plasma miRNAs (miR-141, miR-200a and miR-375) levels were significantly associated with baseline CTC count and that miR-375 was associated with the 28-weeks PSA response. The function of one miRNA can be controversial in different cancers due to tissue-specificity. For instance, miR-21 was consistently identified as an oncomiRNA in various malignancies, including prostate, breast and bladder cancers [91,92,93], whereas miR-125b was reported as oncomiR in PCa and a tumor suppressor in breast cancer [94]. This dual-action suggests the possibility that the same miRNA can participate in distinct pathways to elicit different cellular effects that are dependent on the cell type and target expression in a context-specific fashion.

As miRNAs have several target mRNAs and one mRNA can be regulated by different miRNAs, deregulated miRNA-mRNA networks were found in cancer and can participate in various carcinogenesis-related pathways [92,95,96,97].

Several studies have investigated the role of miRNAs in PCa carcinogenesis and their relationship with the clinical course of the disease as well as their potential role as biomarkers [98,99,100,101] (Table 1), and there is a growing body of evidence indicating that the adaptive communication between cancer cells and their environment (local and distant) is realized through miRNAs-mediated metastatic progression [38].

Different research groups were focused on the correlation between differential expression of specific miRNAs and PCa metastatic burden and aggressiveness, with the ultimate goal to provide potential valuable tools to refine risk stratification and to reduce overtreatment. Bryant et al. reported a significant over-expression of miR-375 and miR-141 in a metastatic cohort compared with a non-metastatic one [102].

Bidarra et al. [103], in a recent exploratory study, confirmed that miR-375-3p expression levels at the diagnosis are an independent predictor for metastasis development, with a 71% specificity. Moreover, high circulating miR-375-3p associate with reduced metastasis-free survival (MFS) not only in high-grade but also in patients with localized, low-grade tumors, allowing to stratify patient groups with quite different clinical outcomes.

The differential expression of miR-141 was correlated with PCa progression [104] in the study by Osipov et al. and used to discriminate between metastatic vs. non-metastatic patients in the study by Li et al. [105]. Finally, a recent publication by Zhao et al. [106] reported how miR-199b-5p down-regulation was associated with metastatic PCa.

Hudson et al. [107] performed a comprehensive differential miRNA expression analysis of a group of PCa patients, finding that the upregulation of the miR-106b-25 cluster is associated with low caspase 7, thus favoring tumor progression and spread.

A distinctive miRNA signature was found in correlation with epidermal growth factor receptor (EGFR) signaling, which controls PCa aggressiveness and progression. In particular, a reduced expression of miR-133, miR-875-5p and miR-146a was significantly related to elevated EGFR signaling and PCa progression [108,109,110].

In a recent work, Bhagirath and colleagues [111] demonstrated how miR-4288, located in a chromosomal locus frequently deleted in PCa progression, could be associated specifically with tumor progression and metastatic burden. Similarly, a study by Ibrahim et al. [112] reported how miR-141, miR-18a, miR-221 and miR-21 expression in the plasma significantly stratified localized from metastatic PCa.

Since 2011, when miRNA-200c was shown as responsible for the transition from oligo to polymetastatic phenotype [32] in a mouse model, miRNAs were considered as valuable candidates for the identification of true oligometastatic patients who would likely benefit from an MDT [113].

The study by Lussier et al. [32] tested miRNA profiles in tissues from oligometastatic and polymetastatic patients. These authors identified a set of miRNAs reflecting the metastatic progression rate in oligometastatic patients treated with SBRT. The same authors validated in two case series their prioritized list of miRNAs and were able to predict metastatic behavior in a homogeneous study in lung cancer patients treated with pulmonary resection [33]. A combined analysis encompassing both the previously cited studies confirmed, notwithstanding the small sample size, the different molecular profiles of the oligometastases and polymetastases, and that miRNA plays a relevant role in the regulation of both these biological conditions [113]. As the miR-200 family is indeed involved in the EMT process [114,115], particular attention was paid to this class of non-coding transcripts as potentially good candidates in oligo PCa prognosis. The miR-200 family includes five members: miR-200a, -200b, -200c, -141, and -429 that play a crucial role in cancer initiation and metastatisation. In particular, inhibition of the members of this family results in increased cell migration; conversely, overexpression of the miR-200 members represses EMT, inhibiting cancer cell motility and migration [114,115].

In contrast to these findings, a 2019 study by Dhondt et al. [31] reported that a multivariate model trained with clinical parameters and serum-derived small RNA sequencing data had no predictive ability to distinguish between OMPC and PMPC cancer patients. The range of expression values between the discovery and validation cohort changed for some of the miRNA targets, and none of the 41 miRNA targets was differentially expressed between oligometastatic and polymetastatic PC patients in the validation cohort.

Therefore, more data are needed to identify and validate a strong miRNA signature for the discrimination between PMPC patients and true oligometastatic patients who could effectively benefit from an MDT.

Despite promising, an accurate measure of miRNAs in biological fluids is very challenging. Technical and pre-analytical factors may have a major influence on miRNA detected levels, thus inducing biases in accurate quantification in biological samples. Therefore, a standard in the procedures for sample collection, handling and storage is of major importance [116,117].

### Epigenetic (epi)-miRNAs

As previously underlined, miRNA are small non-coding RNAs with a major role in gene expression regulation. It was estimated that about 60% of human genes are under the regulatory control of miRNAs [115], and emerging evidence points to a key role of miRNAs as determinants of epigenetic regulation [118,119].

Epigenetics is the study of chromatin modifications, which does not involve alterations in the DNA sequence, but modifications at the level of DNA-scaffold proteins named histones and/or directly involving DNA, such as methylation or alkylation, thus affecting genetic expression. miRNAs can regulate these epigenetic mechanisms at the post-transcriptional level. In particular, the emerging concept of epi-miRNA [120] refers to a class of miRNA that regulates, directly or indirectly, the epigenetic modifiers and could open the way towards novel insights on the role of miRNAs as biomarkers.

The first identified epi-miRNA is the miR-29 family, which seemed to have a direct impact on DNA (cytosine-5)-methyltransferase 3 (DNMT-3) A and B in lung cancer [121]. Few other examples in different tissue types are miR-101 [122] targeting the histone-lysine N-methyltransferase EZH2 or DNMT3b regulation mediated by miR-148a/b [123]. Nevertheless, the number of experimentally validated epi-miRNAs is still very low [124]. A recent study by Gurbuz et al. [125] provided the first evidence of the combined effect of two epigenetic mechanisms, miRNA and DNA methylation, on PCa metastatic progression. Considering the methylation profiles, Gurbuz and colleagues identified 8 miRNAs, among 30 epi-miRNAs analyzed, which are promising biomarkers for the prediction of PCa progression.

These results demonstrate that the integration between miRNA and epigenetic data, with the help of technologies such as ChIP-seq, could unveil novel interactions between miRNA and methylation mechanisms, laying the foundations for the identification of brand new biological linkages and novel prognostic markers [126].

## 6. Conclusions

Further research is needed to evaluate novel biomarkers as promising tools to be implemented in the therapeutic workflow in the oligometastatic setting. Overall scientific evidence analyzed in this narrative review will be applied to the prospective phase II RADIOSA trial [18]. In particular, a deeper understanding of the molecular workings underlying the oligometastatic clinical entity could unravel novel suitable biomarkers that could aid the clinical management of the oligometastatic PCa patient. The most attractive ones are CTCs, cf DNA and miRNA, with technologies such as liquid biopsies and NGS expected to play an important role in the clinical setting.

Additional molecular biology research is also needed in order to establish and define consistent isolation and quantification methods for specific biomarkers assessment. In this scenario, different ongoing trials for biomarker identification in PCa [127] (Table 2) or ongoing trials as the phase 2 Oriole trial and the RADIOSA trial [17,18] might provide additional insights on the biology of the oligometastatic state, laying the bases for the identification of new biomarkers for the accurate outlining of true oligometastatic patients. Overall, this could pave the way to a better personalized medicine approach in the OMPC setting.

## Figures and Tables

**Table 1:** Summary of some of the main studies investigating the role of miRNAs in PCa carcinogenesis and their relationship with the clinical course of the disease as well as their potential role as biomarkers.

Author and Year	Samples Analyzed	Significant miRNAs Analyzed	miRNA Modulation	Clinical Value
Cheng et al. 2018 [81]	mPCa (50 pts)	miRNAs miR-141, miR-200a, miR-200c and miR-375	Baseline vs. end of treatment	miR-375 and miR-200b were significantly associated with 28 weeks PSA response miR-141, miR-200a, miR-200c and miR-375 levels were significantly correlated with CTCs levels
Bryant et al. 2012 [93]	Pca (78 pts, including mPCa and lPCa pts) and normal control (28 pts)	miR-375 and miR-141	Metastatic PCa vs. localized PCa	↑ miR-375 and miR-141 expression significantly increased in metastatic Pca
Li et al. 2016 [96]	PCa (20 pts), BPH (20 pts), Healthy individuals (20 pts)	miR-141	PCa vs. BPH vs. healthy	↑ Elevated levels of serum exosomal miR-141 were considerably correlated with cancer metastasis
Osipov et al. 2016 [95]	PCa (48 pts) and Healthy donors (48 pts)	miR-141, miR-205	PCa vs. healthy	↑ The two miRNAs were significantly upregulated in PCa pts. miR-141 expression level efficiently discriminates early-stage prostate cancer patients and correlates with the Gleason score miRNA-205 expression showed no dependence on the stage of PCa
Zhao et al. 2019 [97]	localized PCa (25 pts) mPCa (35 pts) with bone or lymph node metastases metastases	miR-199b-5p	lPCa vs. mPCa	↓ Exosomal miR-199b-5p serves as a tumor suppressor with prognostic impact in human PCa. Down-regulating miR-199b-5p might confer a proliferative advantage, accelerate migration, and promote metastasis in PCa cells
Bidarra et al. 2019 [94]	lPCa and mPCa (350 pts) and Healthy individuals (52 pts)	miR-182-5p and miR-375-3p	PCa vs. lPCa vs. healthy	↑ miR-182-5p and miR-375-3p were associated with more advanced pathological stages. Higher circulating miR-375-3p levels in pts more prone to develop the metastatic disease with 71.43% accuracy.
Hudson et al. [98]	28 non-cancerous tissues, 99 primary tumors and 14 distant metastases	miR-106b-25 cluster	Tumor tissues vs. metastatic tissue vs. non-cancerous tissues	↑ miR-106-25 increased expression associated with PCa progression and disease prognosis, and caspase-7 is identified as a target of this cluster.

↑ Increased expression ↓ decreased expression. **List of abbreviations: BPH** = benign prostatic hyperplasia; **lPCa** = localized PCa; **mPCa** = metastatic PCa; **PCa** = prostate cancer; **RP** = radical prostatectomy.

**Table 2:** Summary of ongoing trials (all in the recruiting phase) for the identification of predictive biomarkers for prostate cancer.

Trial ID	Trial Description	Study Type	Conditions	Interventions	Outcomes Measures	Estimated Primary Completion Date
**NCT04324983**	BioPoP, Identification of Predictive Biomarkers	Interventional	Prostate Cancer Recurrent	Blood sample	-Rate of complete biochemical response -Prostate cancer-specific treatment-free survival after salvage surgery -Questionnaire Quality of life	December 2021
**NCT03902951**	Antiandrogen Therapy and SBRT in Treating Patients With Recurrent, Metastatic Prostate Cancer	Interventional, Phase II	-Metastatic Prostate Adenocarcinoma -Recurrent Prostate Carcinoma	-Drugs: Abiraterone Acetate/Apalutamide/Leuprolide Acetate -Stereotactic Body Radiation Therapy	-Percent of patients achieving a PSA < 0.05 ng/mL -Time to biochemical/ radiographic progression -Time to initiation of alternative antineoplastic therapy -Prostate cancer-specific Survival -Health-related quality of life -Biomarker analysis	July 2021
**NCT03421015**	Genetic Analysis of Prostate Cancer to Identify PredictiveMarkers of Disease Relapse or Metastatic Evolution	Observational Retrospective	Prostate Cancer	-	-The genetic alteration frequencies of TMPRSS2- ERG gene fusion •Frequency of amplification of proto-oncogenes (MYC, AR, PIK3CA) •Frequency of mutations or deletions of tumor suppressor genes (PTEN, TP53, NKX3-1), •Frequency of point mutations modifying protein function	July 2020

## List of Abbreviations

**ADT**	androgen deprivation therapy
**ChIP-seq**	chromatin immunoprecipitation sequencing
**CT**	computed tomography
**cfDNA**	circulating cell-free DNA
**ctDNA**	circulating tumor DNA
**CTC**	circulating tumor cell
**EGFR**	epidermal growth factor receptor
**EMT**	epithelial mesenchymal transition
**EpCAM**	epithelial cellular adhesion molecule
**hTERT**	telomerase reverse transcriptase
**miRNA**	micro Ribo-Nucleic Acid
**MDT**	metastases directed treatment
**NGS**	nextgeneration sequencing
**OCS-PCa**	oligorecurrent-castration-sensitive-PCa
**OMPC**	oligometastatic prostate cancer
**PCa**	prostate cancer
**PCR**	polymerase chain reaction
**PET**	positron emission tomography
**PMPC**	polymetastatic prostate cancer
**PSA**	prostate-specific antigen
**PSMA**	prostate-specific membrane antigen
**RISC**	RNA-induced silencing complex
**SBRT**	stereotactic body radiotherapy
**SNP**	single nucleotide polymorphism
**WB-MRI**	whole-body magnetic resonance imaging
